# Temperament, physical activity and sedentary time in preschoolers – the DAGIS study

**DOI:** 10.1186/s12887-021-02593-4

**Published:** 2021-03-16

**Authors:** Marja H. Leppänen, Kaisa Kaseva, Riikka Pajulahti, Katri Sääksjärvi, Ella Mäkynen, Elina Engberg, Carola Ray, Maijaliisa Erkkola, Nina Sajaniemi, Eva Roos

**Affiliations:** 1 Folkhälsan Research Center, Topeliuksenkatu 20, 00250 Helsinki, Finland; 2 Faculty of Medicine, University of Helsinki, Helsinki, Finland; 3 Cicero Learning, Faculty of Educational Sciences, University of Helsinki, Helsinki, Finland; 4 Department of Sports and Exercise Medicine, Clinicum, University of Helsinki, Helsinki, Finland; 5 Department of Food and Nutrition, University of Helsinki, Helsinki, Finland; 6 Department of Education, University of Helsinki, Helsinki, Finland; 7 Department of Psychology and Logopedics, Faculty of Medicine, University of Helsinki, Helsinki, Finland; 8 Philosophical Faculty, School of Applied Educational Science and Teacher Education, University of Eastern Finland, Joensuu, Finland; 9 Department of Public Health Clinicum, University of Helsinki, Helsinki, Finland

**Keywords:** Behavior styles, Exercise, Pediatrics, Physical activity recommendation, Sedentariness

## Abstract

**Background:**

Identifying individual characteristics linked with physical activity (PA) and sedentary time (SED) can assist in designing health-enhancing interventions for children. We examined cross-sectional associations of temperament characteristics with 1) PA and SED and 2) meeting the PA recommendation in Finnish children.

**Methods:**

Altogether, 697 children (age: 4.7 ± 0.9 years, 51.6% boys) within the Increased Health and Wellbeing in Preschools (DAGIS) study were included. Parents responded to the Very Short Form of the Children’s Behavior Questionnaire consisting of three temperament dimensions: surgency, negative affectivity, and effortful control. PA and SED were assessed for 7 days (24 h per day) using a hip-worn ActiGraph accelerometer, and the daily minutes spent in light PA (LPA), moderate PA (MPA), vigorous PA (VPA), and SED were calculated. The PA recommendation was defined as having PA at least 180 min/day, of which at least 60 min/day was in moderate-to-vigorous PA. Adjusted linear and logistic regression analyses were applied.

**Results:**

Surgency was associated with LPA (B = 3.80, *p* = 0.004), MPA (B = 4.87, *p* < 0.001), VPA (B = 2.91, *p* < 0.001), SED (B = − 11.45, *p* < 0.001), and higher odds of meeting the PA recommendation (OR = 1.56, *p* < 0.001). Effortful control was associated with MPA (B = − 3.63, *p* < 0.001), VPA (B = − 2.50, *p* < 0.001), SED (B = 8.66, *p* < 0.001), and lower odds of meeting the PA recommendation (OR = 0.61, *p* = 0.004). Negative affectivity was not associated with PA, SED, or meeting the PA recommendation.

**Conclusion:**

Children’s temperament should be considered when promoting PA in preschoolers. Special attention should be paid to children scoring high in the temperament dimension effortful control.

## Background

Physical activity (PA) has been found to contribute favorably to numerous health indicators in preschool-aged children (3–5 years) [1]. Regular PA has been connected, for example with lower adiposity, improved cardiometabolic health, and better cognitive and motor skills development [1]. Sedentary time (SED), however, has been identified as the fourth leading risk factor for noncommunicable diseases by the World Health Organization (WHO) [2]. In accordance with the WHO’s recently updated PA recommendation, preschool children should spend at least 180 min daily at any PA intensity of which at least 60 min should be moderate-to-vigorous PA (MVPA) [3]. Since only a small proportion of preschool children have been reported to comply with the recommendation [4, 5], further clarification of factors related to the compliance of PA recommendations is warranted. As healthy PA habits are established in early life [6, 7], it is important to pay attention to childhood as a critical period with respect to developing the course of health behaviors. Further, it is important to identify factors that potentially play a role in the effectiveness of health behavior interventions initiated at an early age [8].

Regarding intraindividual factors, temperament has shown to be one of the most essential factors contributing to the development of health behaviors from an early age [9]. Temperament is a biologically based, partly inherited behavioral tendency that comprises the core of one's personality [10–12]. Rothbart’s [11] approach emphasizes motor and emotional reactivity as well as attentional processes as regulators of a person’s reactive tendencies. According to this approach [11], the three broad dimensions constitute temperament: 1) surgency (e.g., activity level, sociability, and enjoyment expressed during high-intensity activities), 2) negative affectivity (e.g., anger, sadness, fear, physical discomfort, and recovery from distress), and regulatory capacity in infants and 3) effortful control in older individuals (e.g., ability to focus attention, demonstration of satisfaction during low-intensity activities) [9, 11]. Each of these dimensions may be relevant in the development of health behaviors, such as PA and SED, from an early age [9]. Thus, understanding more deeply temperament’s role in these behaviors may help in supporting children to better manage their potential temperament-related challenges and identify children at higher risk for negative outcomes caused by insufficient PA and increased SED [9]. Furthermore, it is important to recognize the potential benefits of specific behavioral tendencies with respect to the development of a physically active lifestyle. Yet, the existing evidence regarding how the temperamental differences contribute to PA or SED is limited and warrants further research.

To the best of our knowledge, there are only a few previous studies investigating the associations of temperament with PA and SED in preschool-aged children. The majority of the cross-sectional studies have based PA assessment on questionnaires [7–9] or has covered only childcare hours [10], leading to inconsistent findings. A longitudinal study by Schmutz et al. [13] covered a whole day and they reported that temperamental activity [referring to the need for moving, the amount of energy an individual uses for his or her actions, and the speed of his or her behaviors [14]] was positively associated with accelerometer-derived total PA and MVPA and negatively associated with SED. However, as the recently updated global PA recommendation includes PA at any intensity [2], there is a need to study associations of temperament with all PA intensities. Such knowledge would benefit professionals in designing and implementing recreational activities that fit each individual’s temperament [12].

The aims of this study were to investigate associations of temperament dimensions (i.e., surgency, negative affectivity, and effortful control) with I) light PA (LPA), moderate PA (MPA), vigorous PA (VPA), and SED and II) meeting the PA recommendation in Finnish children aged 3–6 years.

## Methods

### Study design and participants

The present study utilizes cross-sectional data from the Increased Health and Wellbeing in Preschools study (DAGIS), which aimed to diminish socioeconomic differences in preschool children’s energy balance-related behaviors [15]. The study was conducted in early childhood education and care (ECEC) centers in southern and western Finland in 2015–2016. The eligibility criteria for the study were: I) having at least one group consisting of 3–6-year-old children, II) providing early education only during the daytime, III) being Finnish or Swedish speaking (official languages of Finland), and IV) being municipality driven. In total, 864 children (25% of the invited children, boys 52%) and their families from 66 ECEC centers (43% of the invited ECEC centers) in 8 municipalities agreed to participate in the study. All children with complete data on temperament and accelerometer data (including ≥3 weekdays and ≥ 1 weekend day) were included in the current study (*N* = 697, 81% of the total sample). Parents/caregivers gave their written informed consent. The study was approved by the University of Helsinki Ethical Review Board in the Humanities and Social and Behavioral Sciences in February 2015 (#6/2015).

### Data collection

Children’s temperament was evaluated using the Very Short Form of the Children’s Behavior Questionnaire that was developed for children aged 3–8 years [16]. One parent in each family indicated their opinion on the 36 items included in the questionnaire, using a 7-point Likert scale ranging from 1 (= extremely false) to 7 (= extremely true). The three broad temperament dimensions established by the instrument developers were constructed from the questionnaire (12 items in each): surgency, negative affectivity, and effortful control. Surgency refers to tendencies to exhibit impulsivity, enjoy situations with high stimulus intensity, and not show discomfort in social situations. Negative affectivity refers to tendencies to have a lowered mood, being angry and fearful, and having difficulties soothing. Effortful control refers to tendencies to have the capacity to suppress inappropriate responses, better self-regulation, and ability to maintain focus on task-related activities [16]. Examples of the questions in each dimension have been previously published [17]. The questionnaire has been shown to demonstrate acceptable internal consistency and criterion validity in children [16]. In the present study, the Cronbach’s alpha values for surgency, negative affectivity, and effortful control were 0.80, 0.76 and 0.74, respectively.

PA and SED were measured using a hip-worn ActiGraph wGT3X-BT accelerometer (Pensacola, FL, USA) for 7 days, 24 h per day. Parents reported the non-wearing hours of the accelerometers (e.g., during water-based activities) in a diary. A 15-s epoch length was used, and periods of ≥10 min of consecutive zeros were regarded as non-wearing time [18]. A valid day was defined as ≥600 min of awake wearing time. The time spent in different PA intensities and SED were calculated using Butte’s cut-points [19], and they were further defined as LPA, MPA, VPA, and SED. The PA recommendation was defined as having at least 180 min of daily PA, of which at least 60 min/day was MVPA [3].

To further test the linkages of temperament with PA and SED, potential confounders (children’s physical characteristics, parental educational level, and the season of the year) were adjusted for in the analyses. Children’s age and sex were reported by the parents. Weight and height were measured by trained researchers, and thereafter, body mass index (BMI) was calculated as body weight (kg) / height^2^ (m). The BMI standard deviation score (BMI-SDS) was computed by the national references [20]. The threshold for being overweight was defined using the age- and sex-specific BMI cutoffs of the International Obesity Task Force criteria [21]. The highest educational level in the household was used as an indicator of socioeconomic status (SES), since SES has been previously associated with PA in children [22]. The educational level of both parents was inquired by a questionnaire, and the higher educational level was further categorized as low educational level (i.e., comprehensive, vocation, or high school), middle educational level (i.e., bachelor’s degree or college), or high educational level (i.e., master’s degree or licentiate/doctor). Seasonal variabilities in weather and daylight may also influence children’s PA and SED behaviors [23]. Since families participated in the study during different seasons, we divided the research season into three categories: fall (September–October), winter (November–December), and spring (January–April).

### Statistical methods

Descriptive information is given as arithmetic means and standard deviations (SD) or frequencies and percentages (%). Using linear regression analysis, we examined associations of each temperament dimension with SED, LPA, MPA, and VPA adjusting for: I) accelerometer awake wearing time, II) child’s age and sex, research season, SES, and awake wearing time, and III) child’s age and sex, research season, SES, awake wearing time, and the two other temperament dimensions. Using logistic regression analysis, we examined associations between temperament and meeting the PA recommendation adjusted similar to the 3 models mentioned above. Sex comparisons among average values were made by using an independent *t*-test for continuous variables and a chi-square test for categorized variables. Statistical tests were conducted using the two-sided 5% level of significance and performed using SPSS Statistics 25 (IBM, Armonk, NY, USA). Although we are presenting cross-sectional analyses, we considered that a sample size of 694 provides 80% power (two-tailed and α = 0.05) to detect an unstandardized regression coefficient of 0.11.

## Results

Table 1 shows the characteristics of the 697 participating children subdivided by sex. Boys were taller and heavier, and they had higher scores in surgency and lower scores in effortful control than girls. Boys also spent less time in SED and more time in MPA and VPA than girls.

**Table 1:** Descriptive characteristics of participating children

	All		Boys		Girls		***P***^***f***^
	N	Mean ± SD	N	Mean ± SD	N	Mean ± SD	
**Age (years)**	697	4.7 ± 0.89	360	4.7 ± 0.88	337	4.7 ± 0.91	0.41
**Height (cm)**	662	109.4 ± 7.87	334	110.4 ± 7.93	328	108.5 ± 7.70	**0.002**
**Weight (kg)**	660	19.1 ± 3.45	333	19.5 ± 3.57	327	18.7 ± 3.29	**0.005**
**BMI-SDS**^**a**^ **(kg/m**^**2**^**)**	661	−0.05 ± 0.98	334	− 0.05 ± 1.00	327	−0.06 ± 0.95	0.94
**Overweight or obese**^**b**^ **(N, %)**	661	77 (11.6)	334	37 (11.1)	327	40 (12.2)	0.64
**Parental education level**^**c**^ **(N, %)**	694		360		334		0.29
Low		152 (21.9)		81 (22.5)		71 (21.3)	
Middle		297 (42.8)		144 (40.0)		153 (45.8)	
High		245 (35.3)		135 (37.5)		110 (32.9)	
**Research season**^**d**^ **(N, %)**	697		360		337		0.76
Fall		308 (44.2)		160 (44.4)		148 (43.9)	
Winter		248 (35.6)		131 (36.4)		117 (34.7)	
Spring		141 (20.2)		69 (19.2)		72 (21.4)	
**Temperament (7-point Likert scale)**							
Surgency	697	4.7 ± 0.86	360	4.8 ± 0.83	377	4.6 ± 0.88	**0.007**
Negative affectivity	697	3.7 ± 0.86	360	3.7 ± 0.85	377	3.7 ± 0.88	0.66
Effortful control	697	5.2 ± 0.73	360	5.0 ± 0.72	377	5.4 ± 0.68	**< 0.001**
**PA and SED (min/day)**							
Awake wearing time	697	773.7 ± 34.38	360	774.6 ± 33.75	377	772.7 ± 35.06	0.45
SED	697	382.9 ± 45.00	360	377.7 ± 44.24	377	388.5 ± 45.20	**0.001**
LPA	697	304.5 ± 31.63	360	304.6 ± 31.62	377	304.5 ± 31.58	0.95
MPA	697	61.3 ± 16.16	360	65.4 ± 15.79	377	56.9 ± 15.39	**< 0.001**
VPA	697	24.4 ± 11.28	360	26.5 ± 12.02	377	22.2 ± 9.98	**< 0.001**
**Meeting the PA recommendation**^**e**^ **(N, %)**	697	586 (84.1)	360	322 (89.4)	377	264 (78.3)	**< 0.001**

*BMI-SDS* body mass index standard deviation score, *LPA* light-intensity physical activity, *MPA* moderate-intensity physical activity, *PA* physical activity, *SED* sedentary time, *VPA* vigorous-intensity physical activity

^a^ According to Saari et al. [20]

^b^ According to Cole and Lobstein [21]

^c^ Low educational level included comprehensive, vocation, or high school; middle educational level included bachelor’s degree or college; and high educational level included master’s degree or licentiate/doctorate

^d^ Fall was defined as September–October, winter was defined as November–December, and spring was defined as January–April

^e^ The PA recommendation was defined as having PA at least 180 min/day of which at least 60 min/day in moderate-to-vigorous PA

^f^ *T*-test for continuous variables and chi-square test for categorized variables. Significant values are bolded

### Associations of temperament with SED and PA

Table 2 presents all three models. A higher score in surgency was associated with lower SED (B = − 11.5, *p* < 0.001) and greater LPA (B = 3.8, *p* = 0.004), MPA (B = 4.9, *p* < 0.001), and VPA (B = 2.9, *p* < 0.001) after adjusting for child’s age and sex, SES, research season, and awake wearing time of the accelerometer (i.e., model 2). After including the other temperament dimensions in the model (i.e., model 3), the associations remained significant (SED: B = − 10.5, *p* < 0.001; LPA: B = 3.5, *p* = 0.014; MPA: B = 4.5, *p* < 0.001; VPA: B = 2.7, *p* < 0.001, respectively).

**Table 2:** Linear regression analysis showing the associations of temperament with sedentary time and physical activity

	Surgency			Negative affectivity			Effortful control		
	***B*** (95% CI)	***β***	***P***	***B*** (95% CI)	***β***	***P***	***B*** (95% CI)	***β***	***P***
**Model 1**									
SED	−11.84 (− 15.38 to −8.30)	− 0.23	**< 0.001**	0.60 (− 3.03 to 4.22)	0.01	0.75	9.43 (5.17 to 13.7)	0.15	**< 0.001**
LPA	3.68 (1.16 to 6.20)	0.10	**0.004**	− 0.84 (− 3.36 to 1.68)	− 0.02	0.52	− 2.60 (− 5.59 to 0.40)	− 0.06	0.089
MPA	5.22 (3.92 to 6.53)	0.28	**< 0.001**	− 0.15 (− 1.50 to 1.21)	− 0.01	0.83	−4.21 (− 5.79 to − 2.63)	− 0.19	**< 0.001**
VPA	3.04 (2.10 to 3.98)	0.23	**< 0.001**	0.36 (− 0.60 to 1.32)	0.03	0.46	−2.69 (− 3.82 to − 1.56)	− 0.17	**< 0.001**
**Model 2**									
SED	−11.45 (− 15.00 to − 7.90)	− 0.28	**< 0.001**	1.20 (− 2.44 to 4.84)	0.02	0.52	8.66 (4.23 to 13.08)	0.14	**< 0.001**
LPA	3.80 (1.24 to 6.37)	0.10	**0.004**	−0.82 (−3.39 to 1.76)	− 0.02	0.53	−2.62 (−5.77 to 0.54)	− 0.06	0.10
MPA	4.87 (3.64 to 6.11)	0.26	**< 0.001**	−0.58 (−1.87 to 0.70)	− 0.03	0.38	−3.63 (−5.18 to − 2.07)	−0.16	**< 0.001**
VPA	2.91 (1.99 to 3.83)	0.22	**< 0.001**	0.13 (−0.81 to 1.07)	0.01	0.79	−2.50 (−3.64 to −1.37)	−0.16	**< 0.001**
**Model 3**									
SED	−10.51 (− 14.28 to −6.74)	−0.20	**< 0.001**	− 0.84 (−4.48 to 2.80)	− 0.02	0.65	5.25 (0.74 to 9.77)	0.09	**0.023**
LPA	3.45 (0.71 to 6.19)	0.09	**0.014**	−0.14 (−2.78 to 2.50)	− 0.00	0.92	−1.52 (−4.80 to 1.75)	−0.04	0.36
MPA	4.47 (3.16 to 5.78)	0.24	**< 0.001**	0.29 (−0.98 to 1.55)	0.02	0.65	−2.18 (−3.75 to −0.61)	− 0.10	**0.007**
VPA	2.71 (1.74 to 3.68)	0.21	**< 0.001**	0.64 (−0.29 to 1.58)	0.05	0.18	−1.60 (−2.77 to −0.44)	− 0.10	**0.007**

The unstandardized regression coefficients (B) with its 95% confidence interval (CI), standardized regression coefficient (β), and *P* value are given for each association. Significant values are bolded

Model 1 was adjusted for awake wearing time, *N* = 697

Model 2 was adjusted for child’s age and sex, research season, socioeconomic status, and awake wearing time, *N* = 694

Model 3 was adjusted for child’s age and sex, research season, socioeconomic status, and awake wearing time and additionally for other temperament dimensions, *N* = 694

*SED* sedentary time, *LPA* light-intensity physical activity, *MPA* moderate-intensity physical activity, *VPA* vigorous-intensity physical activity

A higher score in effortful control was associated with greater SED (B = 8.7, *p* < 0.001) and lower MPA (B = − 3.6, *p* < 0.001) and VPA (B = − 2.5, *p* < 0.001) after adjusting for child’s age and sex, SES, research season, and awake wearing time of the accelerometer (i.e., model 2). After including the other temperament dimensions in the model (i.e., model 3), the associations remained significant (SED: B = 5.3, *p* = 0.023; MPA: B = − 2.2, *p* = 0.007; VPA: B = − 1.6, *p* = 0.007). No significant relationships between negative affectivity and SED or any of the PA intensities were discovered.

### Associations between temperament and meeting the PA recommendation

A higher surgency was associated with higher odds of meeting the PA recommendation after adjusting for child’s age and sex, SES, research season, and awake wearing time of the accelerometer (OR = 1.56, *p* < 0.001) (Table 3). The association remained significant after adding the other temperament dimensions in the model (OR = 1.48, *p* = 0.004). A higher effortful control was associated with lower odds of meeting the PA recommendation after adjusting for child’s age and sex, SES, research season, and awake wearing time of the accelerometer (OR = 0.61, *p* = 0.004), and the association remained significant after adjusting for the other temperament dimensions (OR = 0.67, *p* = 0.026). No significant relationship between negative affectivity and meeting the PA recommendation was discovered.

**Table 3:** Logistic regression analysis showing the associations between temperament and meeting the PA recommendation

	Surgency		Negative affectivity		Effortful control	
	OR (95% CI)	***P***	OR (95% CI)	***P***	OR (95% CI)	***P***
**Model 1**						
Meeting the PA recommendation	1.60 (1.26 to 2.03	**< 0.001**	1.02 (0.81 to 1.30)	0.85	0.62 (0.46 to 0.84)	**0.002**
**Model 2**						
Meeting the PA recommendation	1.56 (1.21 to 2.01)	**< 0.001**	0.98 (0.76 to 1.27)	0.89	0.61 (0.43 to 0.85)	**0.004**
**Model 3**						
Meeting the PA recommendation	1.48 (1.13 to 1.93)	**0.004**	1.05 (0.81 to 1.37)	1.37	0.67 (0.47 to 0.95)	**0.026**

The odds ratio (OR) with its 95% confidence interval (CI) and *P* value are given for each association. *PA* physical activity. The PA recommendation was defined as having PA at least 180 min/day, of which at least 60 min/day was moderate-to-vigorous PA. Not meeting the PA recommendation was set as a reference group

Model 1 was adjusted for awake wearing time, *N* = 697

Model 2 was adjusted for child’s age and sex, research season, socioeconomic status, and awake wearing time, *N* = 694

Model 3 was adjusted for child’s age and sex, research season, socioeconomic status, and awake wearing time and additionally for other temperament dimensions, *N* = 694

## Discussion

Our main findings are that a higher score in surgency was associated with greater PA and lower SED, whereas a higher score in effortful control was associated with lower PA and greater SED in a sample of Finnish preschool-aged children. In addition, a higher score in surgency was associated with higher odds of meeting the PA recommendation, while a higher score in effortful control was associated with lower odds of meeting the recommendation. The results support our hypothesis that temperament, as an intraindividual factor, may have an essential role in contributing to the development of PA and SED behavior at a young age.

We found that a higher score in surgency was associated with lower SED; greater LPA, MPA, and VPA; and higher odds of meeting the PA recommendation. Although the associations slightly weakened, they remained significant after taking the two other temperament dimensions (i.e., negative affectivity and effortful control) into account. This result indicates that after adjusting for the afore-mentioned characteristics in young children, a higher score in surgency is associated with all PA intensities (i.e., LPA, MPA, and VPA) and with lower SED. Our findings are in line with the study by Schmutz et al. [13], who found that temperamental activity in preschool children, based on the parent-reported Emotionality, Activity, and Sociability Temperament Survey, was associated with lower SED and greater PA in longitudinal design. These results, together with ours, highlight that although the temperament dimensions have been defined in a different way, temperament characteristics such as high activity level, high-intensity pleasure seeking, and low shyness and impulsivity seem to be associated with greater PA and lower SED. Moreover, to the best of our knowledge, the association between temperament and meeting the PA recommendation has not been previously studied in preschool children. Thus, our finding that preschool children with a higher score in surgency were more likely to meet the PA recommendation extends the current literature. In addition, we found that surgency was associated with greater LPA, which has not been previously reported. These results indicate that children who have characteristics reflecting higher levels of surgency may more likely gain health benefits that are linked with regular PA [1, 3].

A previous longitudinal study in Finland [12] found that high childhood temperamental activity was associated with decreased levels of self-reported PA in adulthood in men but not in women. Similarly, it is possible that childhood characteristics related to surgency may not straightforwardly contribute to favorable PA behavior up to adult age. In light of this understanding, the potential mechanisms between the examined biologically based characteristics and behavioral outcomes in later life is important. Yet, the knowledge on the potential mediators within the association between temperament and PA during the lifespan is scarce. It is possible that temperament characteristics reflecting surgency may also contribute to the development of a physically inactive lifestyle in later life, as the attractive behaviors become more versatile with age. Therefore, young children who have characteristics reflecting higher levels of surgency and are more physically active may need guidance on how to find high-intensity pleasure from PA to gain health benefits in the long term.

A higher effortful control was significantly related to greater SED, lower MPA and VPA, and lower odds of meeting the PA recommendation. It is possible that the children with higher scores in effortful control feel more need to observe the environment and to control their own behavior, leading to more time spent in SED and less in high-intensity PA, such as MPA or VPA. Moreover, it has been suggested that children with higher levels of effortful control may be more able to follow instructions and prohibitions from adults, which may also affect their behavior by reducing their PA [24]. Thus, it is essential to identify these children to be able to support each child’s natural way of being physically active. This may be, for instance, mindfully restricting the amount of stimulus in an environment in order to support the child’s focus on play and movement. Nevertheless, future studies using a longitudinal design to elucidate this association are still needed to be better able to support children’s PA.

No statistically significant associations of negative affectivity with SED or any of the PA intensities were found, which is partly in line with the two previous studies [25, 26]. A cross-sectional study by Innella et al. [25] also reported that negative affectivity was not associated with parent-reported PA in 100 Hispanic preschool children. However, another cross-sectional study by Sharp et al. [26] found that higher negative affectivity was associated with less outdoor play in 3393 children aged 1–5 years. Because of the different study designs, further studies are needed to investigate the association between temperament and accelerometer-derived SED and PA, and to clarify the role of negative affectivity in PA in young children.

It has been suggested that temperament is a relatively stable construct (e.g. [27]), and each individual’s inherited behavioral styles interact dynamically with the environment across the life span. Therefore, it would be interesting to study whether and how children with different temperamental characteristics establish and maintain PA habits when environmental surroundings (e.g., social referents such as peers) change. Such changes are likely to occur when an individual proceeds through distinct developmental phases (e.g., a transition from adolescence to adulthood). It has been shown that as healthy people mature, they become more self-directed and cooperative [28], which may also associate with the formation of their PA behaviors in later life.

The findings of this study should also be discussed in relation to adults living close to the children and having impact on them. It would be useful that parents become aware of their child’s or children’s temperamental characteristics in order to be able to support their PA in everyday life. Furthermore, it is essential that ECEC centers’ personnel and health care professionals are aware of children’s different temperamental characteristics and their contribution to behavior. This knowledge may help in targeting effective actions to improve PA in children with different reactive tendencies and needs. For instance, in collaboration with other health care professionals (e.g., sports psychologists) pediatricians could design and recommend different types of physical activities for preschoolers. These activities could also vary in terms of their frequencies and intensities depending on the child’s temperamental characteristics. The current study focused on associations of temperament with PA and SED, but previous studies have shown that temperament is also associated with eating habits and screen time in preschoolers [17, 29]. Thus, temperament seems to play an important role in the formation of health behaviors during childhood and should therefore be acknowledged by families and child care professionals in supporting children’s development. The strength of the present study is a relatively large sample size. Moreover, we used accelerometer-derived PA instead of a parent-reported questionnaire to better be able to capture children’s intermittent patterns of movement [18] and the assessment of PA over the entire day. The Very Short Form of the Children’s Behavior Questionnaire was used to evaluate children’s temperament and it has been reported to demonstrate acceptable internal consistency and criterion especially in children aged 3–8 years [16].

The study also has some limitations that need to be considered. Firstly, the cross-sectional study design limits the conclusion about causality between the observed associations. Secondly, the questionnaire used to assess children’s temperament included only three dimensions. Therefore, our results should be confirmed using questionnaires that divides temperament into multiple dimensions to investigate the relationships with PA and SED more deeply. Furthermore, in our study the temperament questionnaire was filled in by only one parent which may lead to misreporting. It has been previously noted that there is non-invariance between mothers’ and fathers’ ratings considering their child’s temperament characteristics [30]. Finally, because of the relatively low participation rate (25%), the sample may be somewhat selected limiting the generalizability of the results into the whole Finnish population.

The statistically significant associations presented in this study raise a need to address their clinical relevance. The magnitudes of the detected associations (i.e., adjusted coefficients) indicate that the findings may be relevant from a clinical point of view. For instance, a Swedish research group has reported that 5 min/day increase in MVPA was longitudinally associated with a healthier body composition and better physical fitness in preschool-aged children [31]. Thus, the results of the current study indicate that the observed associations may be strong enough to have public health importance, and therefore, should be noted when planning future intervention studies.

In conclusion, higher surgency was associated with greater PA, lower SED, and higher odds of meeting the PA recommendation in a sample of Finnish preschool children. Furthermore, higher effortful control was associated with lower PA, greater SED, and lower odds of meeting the PA recommendation. These observational results suggest that one-size-may-not-fit-all and children’s temperament should be taken into account in promoting PA in young children. Thus, motivating children in PA with respect to their temperament characteristics may be essential in health-enhancing interventions. Different temperamental characteristics should be considered when planning interventions to increase PA or decrease SED among young children, or when encouraging children to be physically active. Special attention should be paid to children with self-regulative tendencies.

## Data Availability

Researchers interested in the data and materials from this study may contact principal investigator Eva Roos, eva.roos@folkhalsan.fi.

## Abbreviations

BMI: Body mass index
BMI-SDS: Body mass index standard deviation score
CI: Confidence interval
ECEC: Early childhood education and care center
LPA: Light-intensity physical activity
MPA: Moderate-intensity physical activity
MVPA: Moderate-to-vigorous physical activity
OR: Odds ratio
PA: Physical activity
SD: Standard deviation
VPA: Vigorous-intensity physical activity
